# Can We Consider PR Interval to Screen Patients for Cardiac Resynchronization Therapy?

**DOI:** 10.5935/abc.20180224

**Published:** 2018-11

**Authors:** Martino Martinelli Filho

**Affiliations:** Instituto do Coração do Hospital das Clínicas da Faculdade de Medicina da Universidade de São Paulo (InCor - HCFMUSP), São Paulo, SP - Brazil

**Keywords:** Electrocardiology/methods, Heart Failure/complications, Cardiac Resynchronization Therapy, Review

The search for response markers to Cardiac Resynchronization Therapy (CRT) remains intensive. Currently, the main criteria for CRT indication are the QRS morphology and the absence of myocardial fibrosis.^1^

The electrocardiogram remains an important tool for selecting CRT candidates, and new parameters, such as the PR interval, are interesting to discriminate the prognosis in this population. On this issue, we have a meta-analysis study^2^ concluding that the presence of prolonged PR interval is a marker of poor prognosis at baseline.

In clinical practice, these data may surprise clinicians. The common sense is that it is much easier to make adjustments of the atrioventricular interval to obtain the best hemodynamic response,^3^ as well as to ensure a higher rate of effective atriobiventricular resynchronization.^4^

The pathophysiological hypotheses that could justify this worse prognosis remain a challenge for medicine.

However, a critical view of these data is needed. The question of strong clinical interest is “Can the PR interval be used as a selection criterion for CRT indication?”

This doubt cannot be clarified yet, focusing on findings of this systematic review and meta-analysis. The reason is very clear: the analysis did not include a control group with prolonged PR interval in patients not undergoing CRT, to assess its actual benefit.

Therefore, this meta-analysis adds scientific collaboration, but we still have much more to study!
